# Sleep quality as a predictor of learning engagement and academic self-efficacy among college students

**DOI:** 10.3389/frsle.2026.1837613

**Published:** 2026-05-25

**Authors:** Jae Hee Kim

**Affiliations:** Department of Nursing, Daejin University, Pocheon, Gyeonggi, Republic of Korea

**Keywords:** academic self-efficacy, college students, Korean, learning engagement, sleep quality

## Abstract

Sleep disturbances among Korean college students are a persistent and escalating concern that significantly impacts learning performance. This study aimed to examine the association of sleep quality with learning engagement and academic self-efficacy among college students. The study participants were 195 second- to 4th-year students at universities in the Seoul metropolitan area, sampled through convenience sampling. An online survey was conducted from March 12 to April 8, 2025, adhering to ethical standards throughout the process. Data were analyzed using *t*-test, ANOVA, and simultaneous multiple regression analysis. Findings indicated that learning engagement was significantly higher among students with high academic achievement satisfaction, major satisfaction, and college life adjustment. Academic self-efficacy was greater among male students compared to female students, and among students with higher academic achievement satisfaction, major satisfaction, and college life adjustment. Regression analysis results indicated that sleep quality was a significant influencing factor for both learning engagement and academic self-efficacy. These findings suggest that sleep quality may be a modifiable factor worth targeting in future intervention research aimed at supporting learning engagement and academic self-efficacy. When developing these programs, incorporating strategies that consider gender, academic achievement satisfaction, major satisfaction, and college life adjustment may further enhance their effectiveness.

## 1 Introduction

College students are in a transitional period between adolescence and adulthood, navigating intensive academic demands alongside career preparation pressures. These combined burdens can compromise their physical and mental health.

Sleep is a representative indicator of mental health status. When academic burden is excessive, students may experience both quantitative and qualitative sleep problems. Sleep problems among Korean college students appear to be persistent and increasing. According to a meta-analysis, the prevalence of sleep disorders was 35.6% in the studies published before 2010, but rose to 51.4% in the studies published after 2011 (Hwang and Shin, 2020). These studies approached sleep disorders from the qualitative aspects of sleep, including difficulty falling asleep, difficulty maintaining sleep, and waking up too early. Korean college students' sleep quality, as measured by the Pittsburgh Sleep Quality Index (PSQI), averaged 5.9 points, indicating poor sleep quality (Kim et al., 2022). Furthermore, recent surveys show that the average sleep duration for Korean college and graduate students decreased from 8 h and 22 min in 2019 to 8 h and 19 min (Statistics in Korea).

Sleep is a basic human need for survival and plays a vital role in maintaining a healthy life and high quality of life. Conversely, insufficient sleep quantity or deteriorated sleep quality can lead to physical symptoms such as headaches, fatigue, and decreased activity, as well as psychological issues like aggression and depression. Sleep can also influence academic performance. Adequate sleep facilitates consolidation of memory and enhances attention and cognitive functioning. Moreover, sleep plays a crucial role in emotional regulation, such as anxiety and depression, which can directly or indirectly affect academic achievement (Hershner and O'Brien, 2018). A study focusing on transcranial stimulation found that insufficient sleep causes increased brain excitability and plasticity changes, which can lead to cognitive decline (Salehinejad et al., 2022).

Unlike objectively measured sleep quantity, sleep quality is defined as an individual's self-satisfaction with all aspects of the sleep experience (Birco et al., 2025). Higher sleep quality enables more effective recovery from physical and mental fatigue, allowing individuals to perform tasks more efficiently and maintain optimal condition (Birco et al., 2025; Xu et al., 2025). In contrast, low sleep quality reduces learning engagement and negatively affects academic self-efficacy, which is the ability to execute and solve academic tasks. Research on college students has shown that they experience low sleep quality due to examinations and assignments, in turn affecting their academic performance (Kim and Park, 2024). Sleep quality, learning engagement, and academic self-efficacy are closely interconnected, and when one element weakens, a vicious cycle can occur where the other elements are also negatively affected.

Sleep research related to learning has primarily focused on academic performance. A study of first year college students reported that longer nocturnal sleep duration was associated with higher end-of-semester grades (Creswell et al., 2023). Additionally, sleep disorders have been confirmed to affect subjective academic outcomes such as exam failure and learning delays (Vedaa et al., 2019). Learning engagement and academic self-efficacy are concepts that have been identified as factors influencing academic performance. Learning engagement refers to a state where learners concentrate on and actively engage in classes, going beyond simple class participation (Munangatire and Indjamba, 2022). Martin et al. defined learning engagement in distinction from learning motivation: motivation refers to dispositions, energy, emotions, and drive related to learning, effective task performance, and achievement; whereas learning engagement refers to the behaviors that reflect these dispositions, energy, emotions, and drive (Martin et al., 2017). According to previous studies, learning engagement plays a positive role in various aspects, including not only academic performance but also critical thinking and cognitive development, academic persistence, and mental health (Laranjeira and Teixeira, 2025; Peng, 2021).

The present study draws on two complementary frameworks. First, Bandura's (1997) social cognitive theory posits that self-efficacy is shaped by physiological and affective states, to which sleep quality is a salient indicator. Second, the research of Hershner and O'Brien (2018) and Salehinejad et al. (2022) suggest that sleep quality determines the brain's availability of executive resources necessary for sustained learning engagement. Together, these frameworks provide a theoretical basis for expecting that sleep quality is associated with both learning engagement and academic self-efficacy among college students.

Compared to its importance as a modifiable physiological factor to academic functioning, sleep quality remains underexplored. While positive associations between sleep quality and academic self-efficacy have been reported in Turkish and Chinese college student samples (Aydin and Aydin, 2024; Liu, 2022), evidence on learning engagement is largely limited to adolescent populations (e.g., Xu et al., 2025). However, whether sleep quality is simultaneously associated with both learning engagement and academic self-efficacy among college students remains underexplored, particularly in the Korean context where persistently poor sleep quality has been documented. Given that Korean college students persistently report poor sleep quality (Hwang and Shin, 2020; Kim et al., 2022), this population offers a particularly informative context in which to examine these relationships.

Academic self-efficacy refers to an individual's judgment of their ability to organize and execute the actions required to perform academic tasks (Bandura, 1991). Academic self-efficacy functions as a positive psychological variable that prevents academic burnout and academic dropout, leading students with high academic self-efficacy to have high learning participation and improved academic achievement (Meng and Zhang, 2023). Given the critical role of academic self-efficacy in academic performance, it is necessary to examine the influence of sleep quality as a means of enhancing academic self-efficacy.

This study aimed to examine the association of sleep quality with learning engagement and academic self-efficacy among college students, and to propose strategies for enhancing learning engagement and academic self-efficacy through the lens of sleep quality.

## 2 Methods

### 2.1 Research design

This study employed a correlational design to examine the association of sleep quality with learning engagement and academic self-efficacy among college students.

### 2.2 Participants

The target population for this study consisted of 2nd- to 4th-year students enrolled in universities in the Seoul metropolitan area. The accessible population included students from four specific universities. 1st-year students were excluded to account for the timing of data collection, which occurred early in the academic year.

The required sample size of 153 was determined using G^*^Power 3.0.10 for regression analysis. The criteria applied included an effect size of.15, significance level of.05 (two-tailed), power of 0.80, and 7 predictors including anticipated control variables and independent variables. To account for the minimum sample size and anticipated incomplete responses, 200 participants were targeted, resulting in a final sample of 195 students for the analysis.

### 2.3 Data collection methods

Data collection was conducted using anonymous online community platforms at the four participating universities. After receiving a detailed explanation of the purpose of the research and assurance of anonymity, only the participants who provided consent were sent a survey link via Naver Form. Each survey took approximately 10–15 min to complete. The data collection period was from March 12 to April 8, 2025.

### 2.4 Research variables

#### 2.4.1 General characteristics

General characteristics included sex, academic year, academic achievement satisfaction, major satisfaction, and college life adjustment. Academic achievement satisfaction, major satisfaction, and college life adjustment were measured using single item 5-point descriptive rating scales. While the specific validity of these items has not been formally verified, their use is theoretically justified given that these constructs are global, concrete, and singular in nature (Rossiter, 2002). Nevertheless, single-item measures inherently limit reliability estimation and may weaken observed relationships due to increased measurement error.

#### 2.4.2 Sleep quality

Sleep quality among college students was measured using the instrument developed by Yi et al. (2006). This 4-point Likert scale consists of 28 items across 6 factors: daytime dysfunction, restoration after sleep, difficulty falling asleep, difficulty waking up, sleep satisfaction, and difficulty maintaining sleep. Total score ranges from 28 to 112, with higher scores indicating better sleep quality. In Yi et al.'s study, Cronbach's α reliability indicating internal consistency was 0.92, and it was 0.91 in this study.

#### 2.4.3 Learning engagement

Learning engagement was measured using an instrument developed by Suk and Kang (2007) and modified for college students by Lee (2009). It consists of 32 items, with 16 items each in cognitive engagement domain and affective engagement domain. The cognitive engagement domain consists of balance between challenge and ability, integration of action and awareness, clear goals, and specific feedback; while the affective engagement domain consists of sense of control, concentration on tasks, loss of self-consciousness, distortion of sense of time, and autotelic experience. Each item is measured on a 5-point rating scale, with higher total scores indicating higher levels of learning engagement. In Lee's study (2009), Cronbach's α reliability was 0.89, and it was 0.93 in this study.

#### 2.4.4 Academic self-efficacy

Academic self-efficacy among college students was measured using the instrument developed by Kim and Park (2001), comprising 25 items across 3 domains: task difficulty preference (8 items), self-regulated efficacy (10 items), and confidence (7 items). Each item is measured on a 5-point rating scale, with higher total scores indicating higher self-efficacy. In Kim and Park's (2001), Cronbach's α reliability was 0.91, and it was 0.89 in this study.

### 2.5 Data analysis methods

Data were analyzed using SPSS v.29. *T*-tests and ANOVA were used to identify differences in learning engagement and academic self-efficacy according to general characteristics. Pearson correlation coefficients were used to examine relationships between sleep quality, learning engagement, and academic self-efficacy. The associations of sleep quality with learning engagement and academic self-efficacy were examined using simultaneous multiple regression analysis.

The regression model specification, including the selection of covariates and the analytic strategy, was determined prior to analysis based on theoretical considerations and prior empirical literature. For instance, gender was included given documented gender differences in academic self-efficacy (Huang, 2013). Once the potential covariates were chosen, the preliminary difference analyses were conducted to confirm the empirical relevance of these covariates within the present sample. The variables that showed significant differences, then, were entered as covariates to adjust for their potential confounding effects. Categorical covariates were dummy coded with the lowest satisfaction category as the reference group.

### 2.6 Ethical considerations

Participants were provided with explanations regarding the purpose and the content of the research, anonymity, and confidentiality assurance. Participants were informed that their participation was voluntary, that they could withdraw their responses at any time during the survey without any disadvantage, and that the collected data would not be used for purposes other than this research and be destroyed upon the completion of the study. Consent for the survey was obtained electronically via the online survey link. This study was approved in advance by D. University Institutional Review Board (1040656-202412-HR-01-04).

## 3 Results

### 3.1 General characteristics

Out of 195 participants, 154 (79.0%) were female and 41 (21.0%) were male, while 96 (49.2%) were 4th-year students, representing the largest proportion among academic years. Regarding sleep duration, 119 participants (61.0%) reported sleeping 6 h or less per night.

In terms of academic achievement satisfaction, 71 participants (36.4%) responded “disagree” indicating dissatisfaction, which exceeded those who responded “neutral” or “agree,” respectively. On the other hand, for both major satisfaction and college life adjustment, a majority reported satisfaction, with 97 (49.7%) and 134 (68.7%) participants, respectively, responding “agree”. The mean scores for the primary variables were 83.70 (range 55.00–110.00) for sleep quality, 103.44 (range 61.00–160.00) for learning engagement, and 75.27 (range 34.00–114.00) for academic self-efficacy (Table 1).

**Table 1:** General characteristics (*N* = 195).

General characteristics	Categories	*N* (%)
Gender	Female	154 (79.0)
	Male	41 (21.0)
Grade	Sophomore	41 (21.0)
	Junior	58 (29.7)
	Senior	96 (49.2)
Sleeping time	≦6 h	119 (61.0)
	≧7 h	76 (39.0)
Satisfaction of academic achievement	Disagree	71 (36.4)
	Neutral	63 (32.3)
	Agree	61 (31.3)
Major satisfaction	Disagree	37 (19.0)
	Neutral	61 (31.3)
	Agree	97 (49.7)
College life adjustment	Disagree	8 (4.1)
	Neutral	53 (27.2)
	Agree	134 (68.7)
Sleep quality^*^		83.70 ± 12.42 (55.00–110.00)
Learning engagement^*^		103.44 ± 16.45 (61.00–160.00)
Academic self-efficacy^*^		75.27 ± 13.08 (34.00–114.00)

^*^M± SD(range).

### 3.2 Differences in learning engagement and academic self-efficacy according to general characteristics

Analysis revealed that learning engagement differed significantly based on academic achievement satisfaction, major satisfaction, and college life adjustment (Table 2). Learning engagement was higher among those who responded “neutral” or “agree” for academic achievement satisfaction [(*F*_(2, 192)_ = 9.27, *p* < .001)] and major satisfaction [(*F*_(2, 192)_ = 16.66, *p* < .001)]. Learning engagement was also higher among those who responded “neutral” or “agree” for college life adjustment [(*F*_(2, 192)_ = 21.24, *p* < .001)].

**Table 2:** Differences in learning engagement and academic self-efficacy according to general characteristics.

Variables	Learning engagement		Academic self-efficacy	
	M ±SD	*t* (*p*) or *F* (*p*)	M ±SD	*T* (*p*) or *F (p*)
Gender				
Female	101.01 ± 16.28	−0.71 (0.476)	74.23 ± 13.13	−2.16 (0.032)
Male	105.07 ± 17.18		79.15 ± 12.25	
Grade				
Sophomore	99.61 ± 15.07	1.75 (0.177)	73.49 ± 11.79	0.51 (0.603)
Junior	103.09 ± 15.19		76.07 ± 10.78	
Senior	105.29 ± 17.56		75.54 ± 14.80	
Sleeping time				
≦6 hrs	101.73 ± 15.11	−1.83 (0.069)	74.29 ± 11.45	−1.31 (0.191)
≧7 hrs	106.12 ± 18.13		76.80 ± 15.23	
Satisfaction of academic achievement				
Disagree	98.13 ± 13.25	9.27 (< 0.001)	71.06 ± 11.49	7.07 (0.001)
Neutral	103.10 ± 17.45		76.16 ± 13.28	
Agree	109.98 ± 16.68		79.25 ± 13.36	
Major satisfaction				
Disagree	95.32 ± 16.78	16.66 (< 0.001)	71.57 ± 13.18	6.92 (0.001)
Neutral	98.43 ± 13.97		72.11 ± 12.26	
Agree	109.69 ± 15.44		78.66 ± 12.78	
College life adjustment				
Disagree	88.00 ± 13.41	21.24 (< 0.001)	61.25 ± 7.99	12.56 (< 0.001)
Neutral	94.02 ± 14.68		70.42 ± 11.92	
Agree	108.09 ± 15.15		78.02 ± 12.71	

Academic self-efficacy was higher among male students compared to female students [(*t*_(193)_ = −2.16, *p* = 0.032)]. Academic self-efficacy was also higher among students who responded “neutral” or “agree” for academic achievement satisfaction [(*F*_(2, 192)_ = 7.076, *p* = 0.001)]. It was also higher among those who responded “neutral” or “agree” for major satisfaction [(*F*_(2, 192)_ = 6.92, *p* = 0.001)] and college life adjustment [(*F*_(2, 192)_ = 12.56, *p* < 0.001)].

### 3.3 Correlations among variables

Positive correlations were observed between sleep quality and learning engagement (*r* = 0.34, *p* < 0.001), and between sleep quality and academic self-efficacy (*r* = 0.32, *p* < 0.001) (Table 3).

**Table 3:** Correlations between variables.

Variables	Learning engagement	Academic self-efficacy
	*r*(*p*)	
Sleep quality	0.34 (< 0.001)	0.32 (< 0.001)
Learning engagement	1	0.74 (< 0.001)

### 3.4 Sleep quality as a predictor of learning engagement and academic self-efficacy

For each regression analysis, general characteristics identified as significant variables in the difference analysis were included as covariates. Before regression analysis, multicollinearity and autocorrelation were checked, where no issues were found. For multicollinearity, the variance inflation factor (VIF) was used with a criterion of VIF < 10. For autocorrelation, the Durbin–Watson statistic was interpreted against upper critical values at α = 0.01: for the learning engagement model (*k* = 7 predictors, *n* = 195), the upper critical value was *d*_*U*_ = 1.722; for the academic self-efficacy model (*k* = 8 predictors, *n* = 195), *d*_*U*_ = 1.737. In the learning engagement model, VIF ranged from 1.10 to 6.79, and the Durbin–Watson value was 1.84, which exceeds *d*_*U*_ = 1.722, indicating no positive autocorrelation. In the academic self-efficacy model, VIF ranged from 1.03 to 6.80, and the Durbin–Watson value was 2.18, where 4 – 2.18 = 1.82 and exceeds *d*_*U*_ = 1.737, indicating no negative autocorrelation. No violations of multicollinearity or autocorrelation assumptions were found in either model.

The regression model examining the association of sleep quality with learning engagement was statistically significant [(*F*_(7, 187)_ = 10.71, *p* < 0.001)], with an explained variance of 26.0% (Table 4). In this model, sleep quality was identified as a significant predictor of learning engagement [(*t*_(187)_ = 3.54, *p* = 0.001)].

**Table 4:** Regression analysis of the association between sleep quality and learning engagement.

Independent variables(references)	*B*	*S.E*.	β	*C.I*.	*t*	*p*
Satisfaction of academic achievement(disagree)						
Neutral	1.62	2.54	0.05	−3.40−6.64	0.64	0.525
Agree	2.27	2.93	0.06	−3.50−8.05	0.77	0.441
Major satisfaction(disagree)						
Neutral	2.60	3.12	0.07	−3.70−8.61	0.78	0.433
Agree	8.26	3.04	0.25	2.26−14.26	2.72	0.007
College life adjustment(disagree)						
Neutral	0.44	5.73	0.01	−10.88−11.75	0.08	0.940
Agree	9.68	5.70	0.27	−1.56−20.93	1.70	0.091
Sleep quality	0.30	0.09	0.23	0.13−0.47	3.54	0.001
Adjusted *R^2^* = 0.260, [*F*_(7, 187)_ = 10.71, *p* < 0.001].						

The regression model with academic self-efficacy as the dependent variable was also significant [(*F*_(8, 186)_ = 6.02, *p* < 0.001)], with an explained variance of 17.1% (Table 5). In this model, sleep quality was identified as a significant predictor of academic self-efficacy [(*t*_(186)_ = 3.03, *p* = 0.003)].

**Table 5:** Regression analysis of the association between sleep quality and academic self-efficacy.

Independent variables(references)	*B*	*S.E*.	β	*C.I*.	*t*	*p*
Gender(female)						
Male	3.62	2.12	0.11	−0.57−7.81	1.70	0.090
Satisfaction of academic achievement(disagree)						
Neutral	2.66	2.14	0.09	−1.56−6.88	1.24	0.216
Agree	2.50	2.47	0.09	−2.37−7.38	1.01	.312
Major satisfaction(disagree)						
Neutral	−0.90	2.63	−0.04	−6.16−4.21	−0.37	0.711
Agree	2.22	2.56	0.09	−2.84−7.25	0.86	0.389
College life adjustment(disagree)						
Neutral	5.58	4.82	0.19	−3.94−15.09	1.16	0.249
Agree	10.29	4.79	0.37	0.83−19.74	2.15	0.033
Sleep quality	0.22	0.07	0.21	0.08−0.37	3.03	0.003
Adjusted *R^2^* = 0.171, [*F*_(8, 186)_ = 6.02, *p* < 0.001].						

## 4 Discussion

Learning engagement and academic self-efficacy are critical factors that influence college students' academic performance and are, in turn, shaped by various antecedent conditions. This study examined whether sleep quality, which has received limited attention in this context, is associated with these academic outcomes. In the regression analyses, general characteristics identified as significant variables in the difference analysis were included as covariates.

Regarding learning engagement, Pearson correlation analysis indicated that students with higher academic achievement satisfaction, major satisfaction, and college life adjustment demonstrated significantly greater learning engagement, consistent with prior research. For instance, a systematic review and meta-analysis reported that student engagement is positively associated with academic achievement (Wong et al., 2024). Prior research has also shown robust associations between students' academic satisfaction and engagement in higher education contexts (Assefa et al., 2025; Korobova and Starobin, 2015). Additionally, research focusing on nursing students has likewise examined engagement alongside satisfaction-related constructs in contemporary learning environments (Kavuran et al., 2025). Prior research also supports positive associations between students' satisfaction with their overall college experience and engagement-related outcomes (Korobova and Starobin, 2015; Assefa et al., 2025), which aligns with the relationship between college life adjustment and learning engagement observed in this study. While these results are based solely on difference analysis and thus these variables—academic achievement satisfaction, major satisfaction, and college life adjustment—should not be treated as intervention elements, they serve as meaningful considerations when developing strategies to enhance learning engagement.

Regarding the second set of results from the difference analysis, academic self-efficacy was significantly higher among male students than female students and among those with high academic achievement satisfaction, major satisfaction, and college life adjustment. These patterns suggest that satisfaction with academic performance, major, and college life adjustment may be linked to greater confidence in academic abilities. The finding that male college students exhibit higher academic self-efficacy than female college students is consistent with evidence from meta-analytic research on gender differences in academic self-efficacy (Huang, 2013). Furthermore, prior research has identified positive associations between academic self-efficacy and academic performance (Honicke and Broadbent, 2016), and multiple studies grounded in social-cognitive models have linked self-efficacy with academic satisfaction and related satisfaction constructs (Lent et al., 2007; Sheu et al., 2016). Positive relations between academic self-efficacy and college adjustment outcomes have also been documented in longitudinal research with first-year college students (Chemers et al., 2001). These results suggest that enhancing academic self-efficacy within college education requires customized approaches that account for gender differences, and positive experiences related to academic achievement, major, and campus life. However, as with learning engagement, intervention efficiency may be improved by utilizing these general characteristics as participant selection factors, rather than as intervention elements.

Although tangential to the primary focus of this research, a significant positive correlation was observed between the two dependent variables (*r* = 0.74). This robust relationship suggests a high degree of synergy, indicating that learning engagement and academic self-efficacy may be enhanced concurrently through the mediation of sleep quality, rather than as isolated constructs. Furthermore, the mediational impact may exceed the current findings. Beyond the initial influence of sleep quality, the dependent variables likely exert a reciprocal influence, creating a feedback loop that reinforces the overall pattern. As this interaction fell outside the study's primary scope, the underlying mechanisms between learning engagement and academic self-efficacy remained unexplored. Future studies should prioritize investigating these longitudinal influencing pathways.

From regression analyses, sleep quality was identified as a significant predictor of college students‘learning engagement and academic self-efficacy. Specifically, the full model explained 26.0% of the variance in learning engagement, and 17.1% of the variance in academic self-efficacy. While these represent modest-to-moderate effect sizes, they are noteworthy given that sleep quality remained a significant predictor even after adjusting for control variables. These results suggest that higher sleep quality is associated with greater cognitive, behavioral, and emotional engagement in learning activities among college students. While prior studies addressing the relationship between sleep quality and college students' learning engagement are limited, research in similar contexts supports these findings. For instance, a study of high school students confirmed that those with healthy sleep habits participate more actively in academics and exhibited higher motivation (Ybañez et al., 2025). Quality sleep serves as a physiological foundation that facilitates deep engagement by reducing fatigue and maximizing the allocation of attention and cognitive resources during learning (Salehinejad et al., 2022). Beyond fatigue reduction, sleep enhances engagement by promoting memory consolidation, the process through which newly learned information is transferred from short-term to long-term memory (Diekelmann and Born, 2010). Quality sleep enables students to better engage in learning opportunities by facilitating the integration of prior knowledge. Conversely, sleep deprivation can impair critical cognitive functions—including attention, alertness, and judgment—thereby hindering a student's capacity for sustained learning engagement. Given this essential role of sleep, interventions aimed at improving sleep quality warrant investigation as a potential strategy to support learning engagement.

Academic self-efficacy was found to be positively associated with sleep quality, consistent with prior research. A study of Turkish college students reported that sleep quality was positively correlated with academic self-efficacy and cognitive performance (Aydin and Aydin, 2024). Similarly, research involving Korean nursing students reported that sleep quality was positively correlated not only with academic self-efficacy but also with overall quality of life (Lee and Kim, 2023). Furthermore, a recent cross-sectional study of medical students found that poor sleep quality was significantly associated with lower academic performance, further supporting the link between sleep and academic-related outcomes across diverse student populations (Hassan et al., 2025).

Sleep quality may affect college students' ability to perceive their academic abilities positively and to develop beliefs in their capacity to succeed. Poor sleep quality can indirectly reduce academic self-efficacy by impairing cognitive functioning. In addition, poor sleep quality can exacerbate fatigue and stress, leading students to underestimate their abilities and experience academic anxiety, which can further undermine self-efficacy. These patterns may arise because poor sleep quality affects memory, attention, alertness, judgment, decision-making, and overall cognitive abilities, resulting in functional decline and impaired cognitive performance (Hyndych et al., 2025; Khan and Al-Jahdali, 2023). Conversely, high sleep quality can enhance academic self-efficacy by fostering positive self-belief (Mehta, 2022; Simon et al., 2022). Adequate sleep supports mental clarity and positive psychological states, thereby strengthening confidence in one's abilities and capabilities to cope effectively with academic challenges.

The finding that sleep quality had a greater association with learning engagement than with academic self-efficacy (26.0% vs. 17.1%) may be explained by the possibility that active participation and engagement behaviors are more susceptible to immediate physiological and psychological states, including those influenced by sleep quality. In contrast, academic self-efficacy is a relatively stable construct, developed over time through the accumulation of mastery experiences including sustained and successful learning experiences, vicarious learning, and social persuasion (Bandura, 1997), making it less reactive to momentary fluctuations in sleep quality.

However, it should be noted that the cross-sectional nature of this study precludes determining the directionality of these associations. It is also plausible that students who are more engaged in learning or who possess higher academic self-efficacy adopt better sleep practices. Future longitudinal or experimental studies are needed to clarify the temporal and potentially bidirectional nature of these relationships.

While several control variables were significant in the initial difference analyses, only a subset remained significant within the regression models. In the model predicting learning engagement, only academic satisfaction and sleep quality were significant predictors; major satisfaction and college life adjustment were not. In the model for academic self-efficacy, only college life adjustment and sleep quality reached significance, while gender, satisfaction of academic achievement and major satisfaction did not. Given that multicollinearity was not a concern, this discrepancy likely stems from overlapping explanatory power among these variables. Specifically, major satisfaction, academic achievement, and college life adjustment likely function as facets of a broader ‘academic adjustment' construct. Future research should consider modeling these as a single latent variable to better capture their collective influence.

Along with learning engagement, academic self-efficacy is a key determinant of academic achievement. Therefore, improving sleep quality may represent a practical and effective strategy for promoting successful academic outcomes among college students. In implementing such strategies, it is important to recognize that learning engagement and academic self-efficacy vary according to sex, academic achievement satisfaction, major satisfaction, and college life adjustment.

## 5 Conclusion

This study found that sleep quality was significantly associated with both learning engagement and academic self-efficacy. These findings suggest that sleep quality improvement programs warrant investigation as a potential strategy to support learning engagement and academic self-efficacy. For instance, electronic device-based sleep intervention may prove effective, as recent study suggests (Lu et al., 2025). To maximize effectiveness, these programs should be customized to account for student characteristics such as gender, major satisfaction, academic achievement satisfaction, and college life adjustment.

This study has several limitations. First, learning engagement and academic self-efficacy were treated as outcome variables, though they have primarily been addressed as causal variables for academic achievement in previous research. It is suggested that future studies examine academic achievement as the outcome variable, with learning engagement and academic self-efficacy serving potential either moderating or mediating variables. Such analyses were precluded in this study, as academic achievement was measured using a single-item question rather than a validated instrument. Future studies should adopt more thorough justification for the usage, or implement multi-item measures. Second, the participants were recruited through convenience sampling from four universities in the Seoul metropolitan area, which limits the representativeness of the sample. The gender imbalance (79% female) further constrains generalizability. Future research should employ probability sampling across geographically diverse institutions to enhance external validity. Third, the use of cross-sectional design constrained the causal interpretation among variables. Longitudinal research is needed to clarify whether improvements in sleep quality lead to changes in learning engagement and academic self-efficacy.Tables

## Data Availability

The raw data supporting the conclusions of this article will be made available by the author, without undue reservation.
